# Role of the PPAR pathway in atrial fibrillation associated with heart valve disease: transcriptomics and proteomics in human atrial tissue

**DOI:** 10.1038/s41392-019-0093-2

**Published:** 2020-01-17

**Authors:** Huan-Xin Chen, Ming-Yang Li, Yi-Yao Jiang, Hai-Tao Hou, Jun Wang, Xiao-Cheng Liu, Qin Yang, Guo-Wei He

**Affiliations:** 1 Center for Basic Medical Research & Department of Cardiovascular Surgery, TEDA International Cardiovascular Hospital, Chinese Academy of Medical Sciences & Peking Union Medical College, Tianjin, China; 2 Graduate School, Peking Union Medical College, Beijing, China; 3 The First Affiliated Hospital of Zhejiang University, Hangzhou, and School of Pharmacy, Wannan Medical College, Wuhu, China; 4 Department of Surgery, Oregon Health and Science University, Portland, OR USA

**Keywords:** Cardiovascular diseases, Translational research

Dear Editor,

Atrial fibrillation (AF) is one of the most common cardiac arrhythmias. Its prevalence increases significantly with age. It is well known that patients with AF are associated with an increased risk of stroke,^1^ and AF is a great economic burden on the worldwide socioeconomic system. Previous studies have shown that electrical, contractile, and structural remodeling play key roles in the onset and maintenance of AF.^2^ Other possible pathogenesis mechanisms include calcium handling abnormalities, autonomic imbalance, and genetic factors.^3^ However, the exact molecular mechanism associated with AF remains unknown.

Furthermore, heart valve disease (HVD) is associated with an increased risk of thromboembolic events, mainly in patients with AF. A recent report^4^ shows that the prevalence of persistent or permanent AF in HVD is as high as 87.1% in mitral regurgitation and 86.6% in aortic regurgitation. These data demonstrate the particular importance of AF in HVD. However, the molecular mechanism of AF in HVD remains unrevealed, although there was an attempt to discover plasma biomarkers in HVD.^5^

With rapid advances in high throughput technology, it is now feasible to perform large-scale studies on the expression of genes and proteins at the transcription and translation levels using multiomics approaches for a variety of diseases.^6^ In this study, human atrial tissues from patients with HVD in persistent AF preoperatively for at least 6 months or remaining sinus rhythm (as a control) undergoing heart valve replacement surgery were studied by combined transcriptomics and proteomics analyses. The study procedure conforms to the Declaration of Helsinki and was approved by the Institutional Review Board. The tissue was immediately collected from surgery and placed in liquid nitrogen.

The total RNA of the left and right atrial tissue samples (AFLA and AFRA) from the same AF patients and right atrial tissue from SR (SRRA) patients was extracted. Oligo (dT) magnetic beads were used to isolate mRNA from the total RNA. By mixing with fragmentation buffer, the mRNA was then broken into short fragments, and the cDNA was synthesized using the mRNA fragments as templates. Each cDNA library was sequenced in a single lane of the Illumina HiSeqTM 2000 system using paired end protocols according to the manufacturer’s instructions at BGI (Beijing Genomics Institute). The total protein from each sample was extracted, digested with trypsin, and labeled with a unique iTRAQ tag. Because there is a maximum of eight isobaric tags in proteomics analysis by iTRAQ technology, eight tissue samples were allocated to the eight isobaric tags, including LA F patients (AFLA) as well as right atrial tissue (3 AFRA and 2 SRRA) from five patients. Subsequently, 2D-LC-MS/MS analysis was performed to obtain a global overview of the ubiquitous protein expression changes in the samples, and bioinformatics was used for further analysis. The analysis of transcriptomics and proteomics were from the same sample (Table S1). Fold changes >1.2 or <0.8 and *P*-values less than 0.05 were considered significant (Supplementary: Materials and Methods). The flowchart of the study strategy is depicted in Fig. 1a.

**Fig. 1:**
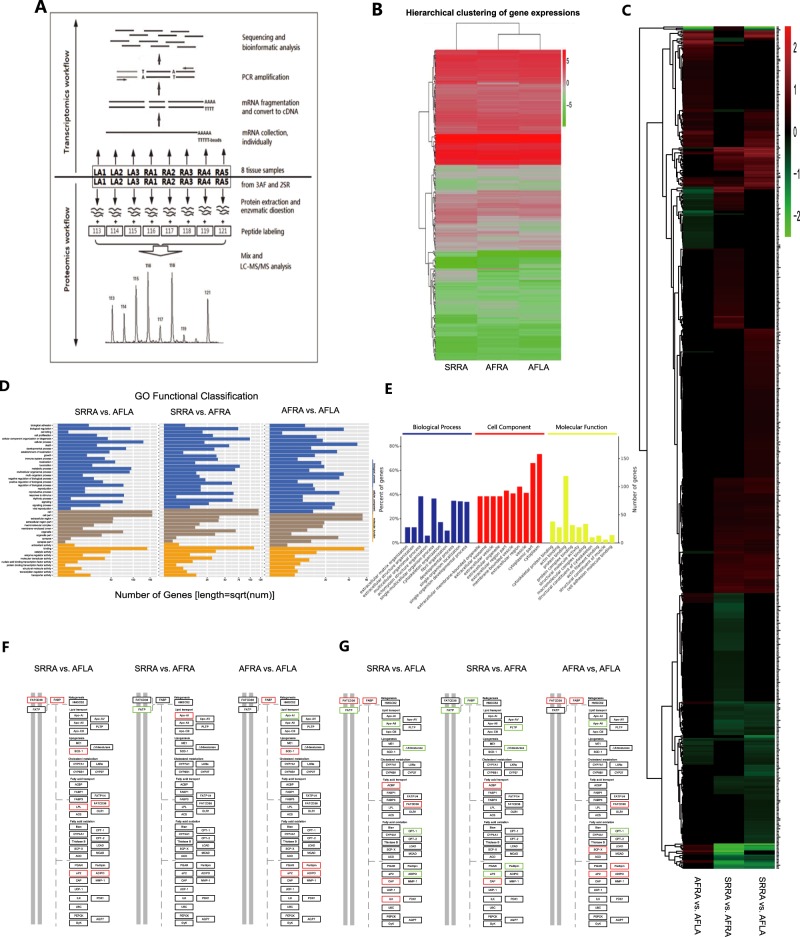
**a** Omics workflow of human atrial tissue samples. **b** Hierarchical clustering of gene expression. **c** Heat map of the proteomics study. **d**, **e** GO functional analysis of differentially expressed genes (**d** comparison between each two of all three groups) and proteins (**e** SRRA vs. AFRA). **f**, **g** Differentially expressed genes identified by transcriptomics (**f**) and proteins identified by proteomics (**g**) involved in the PPAR pathway. Red frames indicate upregulated frames, and green frames indicate downregulated frames.

Big data obtained from the omics studies were analyzed by bioinformatics tools. Hierarchical clustering analysis revealed that the genomic expression of three groups of tissues had differences in transcriptomics profiles (Fig. 1b). Pairwise comparisons were performed between AFLA and SRRA, AFLA and AFRA, and AFRA and SRRA. Differentially expressed genes were identified, including 193 upregulated genes and 279 downregulated genes. A heat map of differentially expressed proteins in the proteomics analysis indicated that there are significant differences in patients with or without AF (AFRA vs SRRA) and between the left and right atrium (Fig. 1c). The details of the differential genes and proteins between the AF and SR patients and between the LA and RA in the AF patients are provided in Table S2.

Gene Ontology (GO) functional and enrichment analysis categorized the differentially expressed genes/proteins in biological processes, cellular components, and molecular functions; most of them participated in biological regulation, immune response, metabolic process, and etc. (Fig. 1d, e).

Most importantly, by using Kyoto Encyclopedia of Genes and Genomes (KEGG) pathway enrichment analysis, we found that the expression of multiple genes and proteins was associated with PPARα, β/δ, and γ, which were altered in both the transcriptomics and proteomics profiles in the atrial tissue of AF patients (Fig. 1f, g). Genes/proteins, such as FATCD36, SCD-1, FABP, ADIPO, APOA1, APOA2, FATP, FADS2, ACBP, CPT-1, aP2, and CAP, are included in or related to the PPAR pathway (Fig. 1f, g). These genes/proteins may be developed as potential biomarkers for disease pathogenesis and progression in AF.

In the mammalian heart, the incessant production of cellular energy is vital for maintaining the continuous mechanical pumping function, providing the body with oxygen and nutrients. To secure the availability of energy substrates, the heart has developed into a “multifuel” organ that is able to use fatty acids, glucose, lactate, amino acids, and ketone bodies as a source of energy. Most forms of cardiac diseases are associated with maladaptive changes in energy metabolism exacerbating disease progression.^7^

The peroxisome proliferator-activated receptors (PPARs, including PPARα, PPARβ/δ, and PPARγ) are a major set of fatty acid-regulated transcription factors controlling lipid metabolism (Fig. S1).^8^ The PPAR signaling pathway regulates the expression of various genes, including proteins involved in fatty acid degradation, glycerophospholipid metabolism, synthesis and degradation of ketone bodies, indicating the central role of PPAR in the metabolic process.^9^ In mitochondria, PPARs regulate energy metabolism by interacting with peroxisome proliferator-activated receptor γ coactivator 1α (PGC-1α), with any point interfered in the PPAR pathway likely to be a causal factor of cardiac pathogenesis.^10^

In summary, our study provides an integrative analysis of transcriptomics and proteomics in the human atrial tissues of AF-associated HVD patients and reveals that the PPAR signaling pathway plays an important role in the pathogenesis of AF and that some proteins may serve as biomarkers for AF. Owing to the importance of the PPAR pathway in the pathology of AF found in heart valve diseases in this study, targeting differential proteins in the PPAR pathway may become new diagnostic and therapeutic methods in the future.

## Supplementary information

Supplementary Materials

## Data Availability

The raw proteome data have been submitted to Proteome X change (Project accession: PXD006911).
