# Supplementary material for: Role of the PPAR pathway in atrial fibrillation associated with heart valve disease: transcriptomics and proteomics in human atrial tissue
Source: Signal Transduct Target Ther. 2020 Jan 17;5:4. doi: 10.1038/s41392-019-0093-2 (PMC6971265; doi:10.1038/s41392-019-0093-2)
Supplement: Supplementary file 1 — Supplementary Materials [file 41392_2019_93_MOESM1_ESM.pdf]

Supplementary Materials for

Role of PPAR Pathway in Atrial Fibrillation Associated with Heart Valvular Disease: Transcriptomics and Proteomics in the Human Atrial Tissue

Huan-Xin Chen[1*], Ming-Yang Li[1,2*], Yi-Yao Jiang[1*], Hai-Tao Hou[1], Jun Wang[1], Xiao-Cheng Liu[1], Qin Yang[1], Guo-Wei He[1,3,4†]

Correspondence to: gwhezj@163.com

This PDF file includes:

Materials and Methods
Figures. S1
Tables S1 to S2

Materials and Methods

Transcriptiomics analysis

The transcriptiomics analysis was performed in the atrial tissue. Five tissue samples from 3 AF patients and 2 SR patients were divided into left atrial tissue group (LA, n=3) and right atrial tissue group (RA, n=3) in the AF patients and right atrial tissue in SR patients (n=2). Tissues from LA and RA in the AF group were obtained from the same patient. Total RNA was extracted from the tissue using TRIzol reagent (Invitrogen, Burlington, ON, Canada) and digested with DNase I (Takara, Dalian, China) according to the manufacturer's protocol. Next, Oligo (dT) magnetic beads were used to isolate mRNA from the total RNA. By mixing with fragmentation buffer, the mRNA was then broken into short fragments. The cDNA was synthesized using the mRNA fragments as templates. The short fragments were purified and resolved with EB buffer for end repair and single nucleotide A (adenine) addition, and then connected with adapters. Suitable fragments were selected for PCR amplifcation as templates. During the quality control steps, an Agilent 2100 Bioanalyzer (Agilent Technologies, Redwood City, CA, USA) and ABI StepOnePlus Real-Time PCR System (Life Technologies, Grand Island, NY, USA) were used for quantifcation and qualifcation of the sample library. Each cDNA library was sequenced in a single lane of the Illumina HiSeqTM 2000 system using paired end protocols according to the manufacturer's instructions at BGI.

Proteomics analysis

A discovery-validation workflow was used in the proteomics analysis. Isobaric tags for relative and absolute quantitation (iTRAQ) technology was used for the proteomics study. Because there are 8 Isobaric tags (m/z113,114,115,116,117,118, 119 and 121), the plasma samples from 24 patients were divided into 6 groups (3 AF group and 3 SR group), each of these groups contained the plasma from 4 patients. In addition, plasma samples from 12 AF or 12 SR patients were used

as control groups, respectively. Thus, the 8 Isobaric tags were concomitantly labeled in the plasma samples for the iTRAQ study.

In the iTRAQ study for atrial tissue, the samples were allocated into groups as described in the transcriptomics study.

In the validation phase, identified differential proteins were validated in a larger study population using enzyme-linked immunosorbent assays (ELISA) in plasma samples.

Protein digestion and iTRAQ labeling

Protein (100 ug) from each sample was precipitated by cold acetone at −20 °C for 1 hour and then centrifuged at 12000rpm for 15 minutes, after which the deposit was collected and dissolved with 50 ul dissolution buffer. Proteins in the solution were reduced (4ul reducing reagent, 60°C for 1 hour), alkylated (2ul cycsteine-blocking reagents, room temperature for 10 minutes), digested (50ul sequencing-grade trypsin (50ng/μL), 37°C for 12 hours) and centrifuged at 12000rpm for 20 minutes. Following digestion with trypsin, iTRAQ regent (AB SCIEX, Framingham, MA, USA) was added and each sample was labeled with a unique iTRAQ tag (m/z113,114,115,116,117,118,119 and 121).

2D-LC-MS/MS analysis

The peptides were dried in a vacuum freeze dryer and re-suspended in 100ul of buffer A. Reversed-phase liquid chromatography (RPLC) was used to fractionate sample on the Agilent 1200 HPLC system (Agilent) equipped with HPLC column (Narrow-Bore 2.1*150 mm 5μm, Agilent). A total of 10 segments were collected at a rate of 0.3ml/min using a nonlinear binary gradient starting with buffer A and transitioning to buffer B. The fractions were dried in a vacuum freeze dryer and re-dissolved in nano-RPLC buffer A. Each segment was loaded on a Eksigent nanoLC-Ultra™ 2D System (AB SCIEX) mounted with a C18 nanoLC trap column

(100 μm ×3cm, C18, 3 μm, 150Å) and separated over a 70 min acetonitrile gradient from 5 to 35% in 0.1% formic acid combined with a Triple TOF 5600 System (AB SCIEX, USA) fitted with a Nanospray III source (AB SCIEX, USA). Data were acquired using an ion spray voltage of 2.5kV, curtain gas of 30PSI, nebulizer gas of 5PSI, and an interface heater temperature of 150 ℃.

iTRAQ data analysis and bioinformatics analysis

After data pre-processing, standardization, and quality control, the original data were screened for reliable peptides and proteins. Identification of peptides from tandem mass spectrometry (MS) was achieved using the Protein Pilot Software v.5.0 (AB SCIEX, USA) against Uniprot_Homo Sapiens Database to match the theory data in order to obtain result of protein identification. The criteria for differential proteins were established as:1) Unique peptide>=1, apart from invalid values and anti-library data; 2) Fold change>1.2 or <0.83; and 3) P value<0.05 (T-test for repeated data for over three times). Proteins fulfilling these criteria were considered to have significant difference in expression between the two groups.

A list of differential proteins identified was implemented on the on-line software DAVID Bioinformatics Resources 6.8 (NIH) for Gene Ontology (including biological process, molecular function and cellular component) function and enrichment analysis. Kyoto Encyclopedia of Genes and Genomes (KEGG) pathway database and STRING database were used to perform pathway analysis and protein-protein interaction analysis.

Data processing and bioinformatics analysis

ChromaTOF software and Fiehn database were used to process the raw data and provide qualitative information about the metabolites. Data were normalized by total peak area of each sample using Excel 2007 and imported into SIMCA-P software (version 14.0, Umetrics, Umeå, Sweden) for multivariate statistical analysis. Principal component analysis (PCA) was used to visualize the general distribution of samples and the stability of the whole analysis process.

Partial least-squares discriminant analysis (PLS-DA) and orthogonal partial least-squares discriminant analysis (OPLS-DA) were used to distinguish the differences of general metabolic profiling between different patient groups. To avoid model overfitting, a default of seven rounds of cross-validation in SIMCA-P software was applied. Moreover, OPLS-DA models were validated by a permutation analysis with 200 times. Differential metabolites were selected according to variable importance in projection (VIP) values>1 in OPLS-DA model as well as P-values<0.05 in Student’s test. KEGG pathway database was used to generate significant pathways in metabolomics dataset.

Creation of pathway maps

All the omics data including transcriptomics, proteomics, and metabolomics data were used to compare proteins/enzymes with metabolic pathways. Quantitative changes of individual metabolites and proteins as compared with control samples were calculated and graphed by using the KEGG mapping tools. In particular, by combining proteomics with metabolomics data, we were able to identify the common pathways involved in atrial fibrillation.

Figure. S1. The differential expression in PPAR pathway in atrial tissues: The overview of PPAR pathway

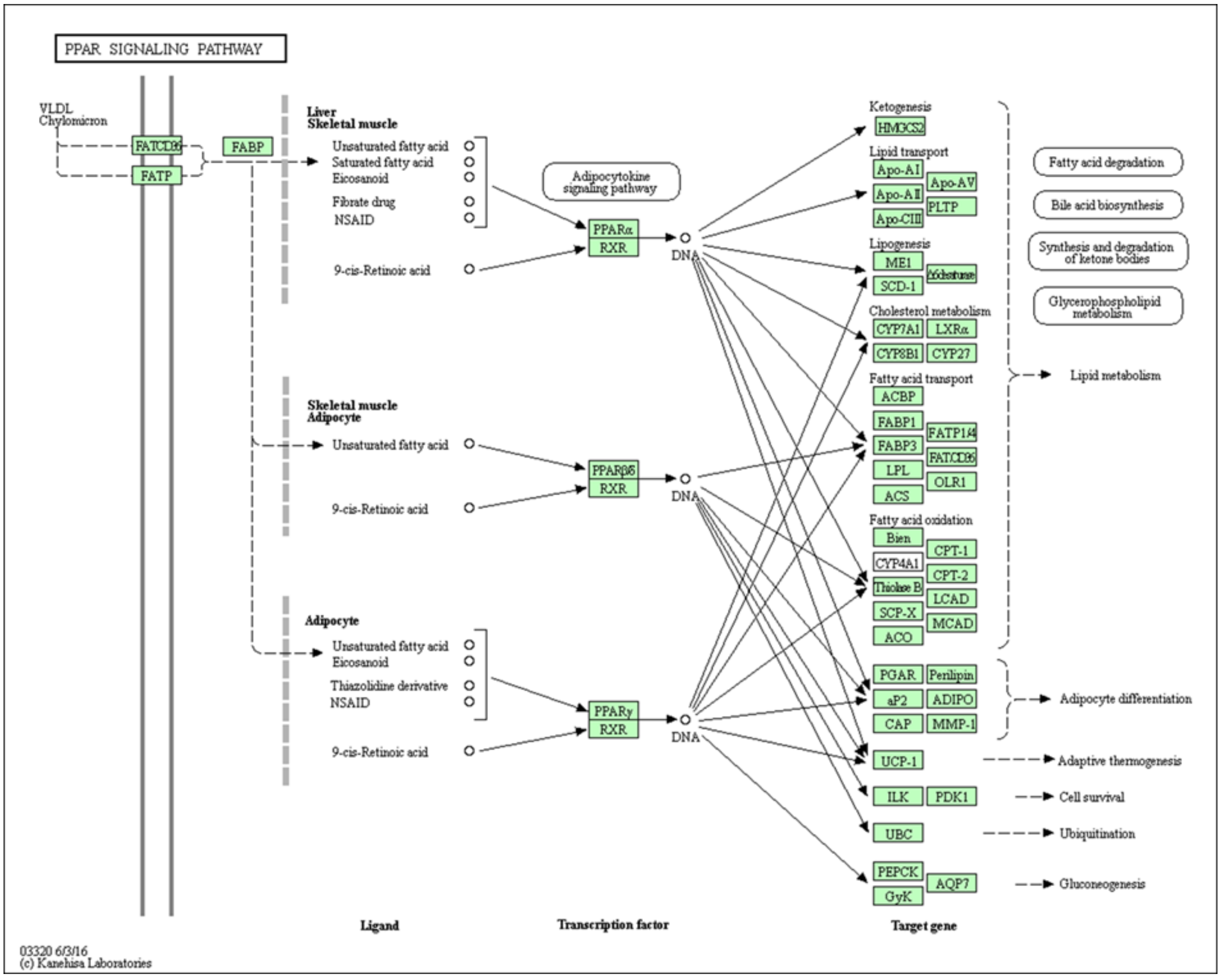

Table S1. Patients Characteristics

Characteristic	AF(n=36)	SR(n=26)
Gender (M:F)	20:16	15:11
Age (year)	59.5±8.2	51.4±13.3
BMI (kg/m^2)	24.0±4.2	23.5±2.6
Hypertension, n (%)	4(11.1)	2(7.7)
Hyperlipemia, n (%)	3(8.3)	1(3.8)
diabetes, n (%)	2(5.6)	1(3.8)
ACE inhibitors, n (%)	2(5.6)	1(3.8)
AT1-blockers, n (%)	0(0)	1(3.8)
Nitrates, n (%)	5(13.9)	2(7.7)
Lipid-lowering drugs, n (%)	3(8.3)	1(3.8)
Ca^{2+}-antagonists, n (%)	2(5.6)	1(3.8)
LV-Dd, mm	55.5±7.2	54.3±8.5
LA-Ds$_{(A-P)}$, mm	53.9±8.0	49.6±6.9
RV-Dd$_{(L-R)}$, mm	36.4±3.2	33.25±3. 5
RA-Ds$_{(L-R)}$, mm	40.4±4.3	36.6±3.1
LVEF	58.4±4.5	62.3±4.3

All variables displayed as mean±SEM;

AF=atrial fibrillation;

SR= sinus rhythm;

BMI= body mass index;

LV-Dd=left ventricular-diastolic diameter;

LA-Ds(A-P)=left atrium-systolic diameter (anterior-posterior);

RV-Dd(L-R)=right ventricular-diastolic diameter (left-right);

RA-Ds(L-R)=right atrium-systolic diameter (left-right);

LVEF= left ventricular ejection fraction;

Table S2. Differential genes and proteins identified in tissues.

A. Differential genes identified in tissues.

SRRA-VS-AFRA		SRRA-VS-AFLA		AFRA-VS-AFLA	
Symbol	log2Ratio	Symbol	log2Ratio	Symbol	log2Ratio
VGLL2	2.756	CFC1	-8.459	DGAT2	1.477
CFC1	-4.344	KRT19	-3.325	PITX2	5.946
KRT19	-3.036	CNTFR	-1.534	GPD1	1.778
CNTFR	-1.522	PLP1	-1.264	IGJ	1.265
CPA4	-2.268	CPA4	-3.013	FOSB	-2.185
PPP1R1A	-1.382	PITX2	5.394	CCL19	1.172
XIST	6.689	GNAO1	-1.253	BMP10	-9.310
LOC100216479	-1.932	XIST	6.455	TUSC5	2.044
ABAT	1.092	DMKN	-2.640	RGS1	-1.701
FNDC5	-1.025	TPSB2	1.539	CLDN5	1.473
PTGDS	-1.200	LOC100216479	-2.830	PRSS35	-2.217
EGR2	2.328	LFNG	1.355	FCGR3A	-1.066
CXCR4	1.167	NDUFA4L2	1.045	ACTG2	1.519
COMP	2.941	COMP	3.230	NPR3	-1.615
UPK1B	-2.086	CGNL1	-1.737	IGLL5	1.680
IGJ	1.444	ASPG	-1.275	NR4A2	-1.565
DNER	-2.951	CD36	1.147	SFRP1	1.335
UPK3B	-4.164	EFHD1	1.397	THBS4	1.822
CD37	1.401	GPD1	1.079	PPP1R14A	1.337
CCL2	1.259	TNFRSF12A	1.908	SMYD2	-1.574
FOSB	3.944	UPK1B	-2.168	CCL21	2.883
PLA2G2A	-1.475	IGJ	2.709	CIDEC	1.801
CCL19	1.763	DNER	-2.515	CIDEA	2.037
CFB	-1.457	GPR126	-1.791	APOD	1.457
CCL4L1	2.058	RGS6	-2.398	ADIPOQ-AS1	1.851
TSIX	4.082	DIRAS3	-1.821	MYOZ1	-2.366
RGS1	3.206	TGM2	-1.065	TAGLN	1.003
RARRES1	-1.372	UPK3B	-3.367	C2orf40	1.178
G0S2	-1.113	CD37	1.343	SLN	-1.897
SULF1	-1.129	CCL2	1.386	DARC	3.484
SMOC1	-1.726	SGK110	-1.349	ART3	-1.056
EFNB3	-2.922	PLA2G2A	-2.037	GPIHBP1	1.660
TNC	1.870	TMEM100	-1.542	SMAD7	-1.044
CD52	2.061	CPNE5	-1.033	TF	1.569
LCP1	1.189	INHA	-2.576	SPP1	-1.316
FHL2	1.479	CCL19	2.936	MYBPHL	1.243
LSP1	1.104	PTN	1.220	CXCL14	2.313
GADD45G	-1.235	CFB	-1.724	CSRP2	1.216
CD79A	3.185	BMP10	-8.817	ADAMTSL2	-1.176
HBG2	-3.787	HCN4	-3.295	ADM	-1.748
PKHD1L1	-1.420	EPHB6	-1.721	MAPK4	-1.092

SOCS3	2.153	TSIX	3.890	MT3	3.051
IGLL5	2.379	RARRES1	-2.099	COLEC11	-1.458
SELENBP1	-1.494	SMOC1	-2.486	PLIN1	1.791
MPZ	-1.540	EFNB3	-2.448	RBP4	1.512
TREM2	1.730	ACTG2	2.189	IGFBP3	-1.091
SPRR2F	-5.383	CD52	2.259	STEAP4	1.177
KLK11	-2.508	LCP1	1.191	ELK2AP	1.421
SOSTDC1	-3.126	CSRP3	1.246	APOA1	-1.518
C19orf33	-2.848	LSP1	1.440	AZGP1	2.027
STXBP6	-1.850	GNLY	2.676	SCD	1.796
RSPO1	-2.961	TCEAL2	-1.607	TENM2	-1.277
ANXA8L2	-2.938	COL6A6	-1.804	TCF15	2.648
CCL3	2.098	A2M	1.112	MYOT	1.461
TGM1	-2.329	HBG2	-4.486	C1orf115	1.359
LMAN1L	-2.533	GSTM5	-1.705	MARCO	-2.572
CCDC163P	-2.547	PKHD1L1	-3.075	CXorf36	1.376
EGR1	1.618	NPR3	-2.027	SMAD6	-1.116
SBSPON	-2.134	IGLL5	4.059	ADIPOQ	1.877
NR4A2	3.541	CACNA2D2	-2.153	FABP4	2.127
ADAMTS1	1.227	SELENBP1	-1.063	MYL2	2.165
PHGDH	-1.774	IRX3	-2.094	LIPE	1.754
SLC6A6	1.288	SPRR2F	-5.447	NTM	-1.706
IGFBP2	2.024	KLK11	-3.511	DNASE1L3	-1.484
C1orf105	-3.340	LTB	1.878	DISC2	-3.050
CRLF1	1.967	SOSTDC1	-2.769	THRSP	1.829
SMYD2	1.097	PENK	3.509	HAMP	-7.334
TM4SF1	-1.179	LRRC49	-1.549	SERF1A	9.442
KRT7	-1.935	CXCL13	8.211	FABP5	1.037
MS4A1	3.678	CPA3	2.441	BTNL9	1.411
CALB2	-1.985	SULT1A3	8.182	CLDN18	4.909
SLC2A3	1.529	C19orf33	-3.284	CHGB	1.024
SUSD4	-1.455	STXBP6	-1.508	FOS	-1.013
PEBP4	-1.034	ANXA8L2	-3.404	ACTA2	1.085
SPOCK2	-1.311	TGM1	-2.444	C1QTNF9	2.610
GAS1	-1.169	LMAN1L	-1.311		
CCL18	1.889	CCDC163P	-2.464		
MYRF	-2.480	SBSPON	-2.516		
RPSAP58	4.477	SFRP1	1.369		
MXRA5	1.612	CD48	1.437		
GALNT16	-1.287	ADAMTS1	1.454		
PDZK1IP1	-3.160	PHGDH	-1.589		
UNC5B-AS1	1.303	HLA-DOA	1.568		
SYT13	-3.518	IGFBP2	2.392		
MSR1	1.436	RPL3L	1.455		
SEMA3B	-1.148	BMX	-1.733		
ATP1B4	3.023	CRLF1	2.216		
PHLDA1	1.531	THBS4	2.501		
MT1L	-1.199	PPP1R14A	1.323		

FCGR2A	1.126	MZB1	6.231
ANXA8	-3.233	MEOX1	1.650
SLC27A6	-1.115	MS4A1	3.322
RARRES2	-1.372	CALB2	-1.503
KRT18	-1.741	CCL21	3.328
HBB	-1.596	THBS2	1.118
CCL4	1.698	CIDEA	1.279
SIK1	2.147	LPL	1.584
CCL3L1	8.458	SUSD4	-2.701
COLQ	2.023	APOD	1.220
CDKN1A	1.458	GAS1	-1.498
BNC1	-2.668	ADIPOQ-AS1	1.594
ITLN1	-3.026	MYOZ1	-2.566
PDGFRL	-1.760	PDPN	-1.651
TNNT1	1.673	MYRF	-2.712
SPP1	3.150	S100A8	1.250
MYBPHL	-1.935	RPSAP58	4.598
MUSTN1	-1.087	GEM	1.436
HBA2	-1.939	GPR22	-1.282
BCYRN1	1.206	PDZK1IP1	-3.313
SFRP5	-2.496	UNC5B-AS1	1.729
MEDAG	-1.437	MFAP5	1.176
JUNB	1.963	SEMA3B	-1.297
GSTM1	-2.721	SLN	-2.811
RBP4	-1.689	ATP1B4	2.640
MYH6	-1.091	PHLDA1	1.429
MAL2	-2.374	DARC	4.241
TNNT3	-1.115	ART3	-2.013
HP	-1.920	ANXA8	-2.901
AOX1	-1.527	TMEM132C	-1.842
PRG4	-2.524	RARRES2	-1.211
TPSAB1	1.939	KRT18	-1.296
SMTNL2	-2.224	HBB	-2.126
SLC7A11	-2.758	COLQ	2.028
CARNS1	-1.364	CDKN1A	1.127
RBP1	-1.254	SMAD7	-1.062
PTPRF	-1.404	BNC1	-3.009
ARL4D	-1.293	ITLN1	-3.667
LBH	1.020	GRIN2C	-1.696
C3	-1.087	TNNT1	1.741
CD83	1.719	SPP1	1.834
NR4A1	1.939	MUSTN1	-1.705
ELK2AP	2.574	HBA2	-2.382
RNA5-8S5	1.699	ADM	-2.064
MT1X	-2.049	BCYRN1	2.146
CPXM1	-1.639	SFRP5	-2.962
UBD	3.926	SLC5A12	-3.500
DHCR24	-1.469	MEDAG	-1.835

APOA1	1.670	PTGS1	-1.777
CLDN15	-2.178	JUNB	1.151
MSLN	-2.965	GSTM1	-2.273
MARCO	-1.514	COLEC11	-2.339
RHBDL3	-2.760	FAM216B	-1.222
SYT4	-2.315	LEPR	-1.174
HBA1	-1.564	MAL2	-2.017
SNORD3A	8.148	HP	-2.608
ANXA8L1	-2.533	AOX1	-1.693
BCHE	-2.013	PRG4	-2.949
GOLGA4	1.197	ITGBL1	1.412
LRRN4	-2.707	SOD3	1.075
ALOX15	-2.776	TPSAB1	2.729
ITLN2	-3.560	SMTNL2	-3.080
C4B_2	-1.424	SLC7A11	-2.542
RCAN1	1.240	DDR1	-1.203
CYP4F30P	-2.550	COL1A1	1.182
OTOGL	-1.666	CARNS1	-2.110
CXCL9	2.836	RBP1	-1.200
MT1G	3.694	ARL4D	-1.808
ASPN	1.289	EMILIN3	-1.419
DHRS9	1.717	CGN	-2.883
DNLZ	1.161	STEAP4	1.268
LYVE1	-1.414	NR4A1	1.285
ZFP36	1.242	ELK2AP	3.996
CFI	-1.100	RNA5-8S5	1.309
HLA-DPA1	1.099	MT1X	-2.384
SHD	-1.965	GPC3	-1.169
CPLX3	-3.102	UBD	3.755
PLLP	-1.941	FAM84A	-4.177
CSRNP1	1.575	AZGP1	1.747
SLPI	-2.226	SCD	1.528
CHGB	2.830	TENM2	-1.286
FOS	1.474	CLDN15	-2.316
ANKRD23	-2.120	MSLN	-3.515
NPPB	4.479	ZDHHC11	-1.247
RPS26	-1.068	MYOT	2.128
		MARCO	-4.086
		CHODL	-3.399
		NOTCH3	1.349
		SYT4	-3.022
		CXorf36	1.448
		HBA1	-2.110
		ADIPOQ	1.686
		SNORD3A	8.486
		ANXA8L1	-3.281
		FABP4	1.694
		BCHE	-1.878

LRRN4	-3.368
MYL2	2.471
NTM	-2.336
SLC4A4	-1.581
ALOX15	-3.481
ITLN2	-3.643
DNASE1L3	-1.504
DISC2	-3.315
VCAN	1.250
RCAN1	1.256
CYP4F30P	-9.194
OTOGL	-1.925
CXCL9	2.357
ASPN	1.185
DHRS9	1.811
S100A9	1.122
SFRP4	1.167
HPR	-1.271
RGS5	1.046
LYVE1	-1.808
ALDH1A2	-1.474
THY1	1.697
HAMP	-7.473
CFI	-1.390
SERF1A	9.442
COL9A1	-2.684
SLC39A8	-1.070
SHD	-1.651
CPLX3	-1.461
PLLP	-2.378
SLPI	-3.065
PRR15	-2.596
CLDN18	4.194
CHGB	3.854
LTBP2	1.334
NPPB	4.553
CYR61	1.586
GPM6A	-2.597

B. Differential proteins identified in tissues.

SRRA-VS-AFRA		SRRA-VS-AFLA		AFRA-VS-AFLA	
Accession	Fold Change	Accession	Fold Change	Accession	Fold Change
tr|I1VZV6|I1VZV6_HUMAN	0.195	sp|O95393|BMP10_HUMAN	0.18	sp|O95393|BMP10_HUMAN	0.116
tr|D3GKD8|D3GKD8_HUMAN	0.243	tr|D3GKD8|D3GKD8_HUMAN	0.205	sp|Q9NP98|MYOZ1_HUMAN	0.294
sp|P02042|HBD_HUMAN	0.245	sp|P02042|HBD_HUMAN	0.316	tr|A0A0A0MSG2|A0A0A0MSG2_HUMAN	0.465
sp|P05787|K2C8_HUMAN	0.258	sp|P08729|K2C7_HUMAN	0.336	tr|B1ALD9|B1ALD9_HUMAN	0.535
sp|P08729|K2C7_HUMAN	0.267	tr|Q5IWS5|Q5IWS5_HUMAN	0.355	sp|Q8TAE6|PP14C_HUMAN	0.555
tr|Q5IWS5|Q5IWS5_HUMAN	0.274	sp|Q9NP98|MYOZ1_HUMAN	0.37	sp|A6NMZ7|CO6A6_HUMAN	0.566
tr|E5RHP7|E5RHP7_HUMAN	0.306	sp|P05787|K2C8_HUMAN	0.382	tr|H3BRN4|H3BRN4_HUMAN	0.579
sp|P00918|CAH2_HUMAN	0.313	tr|A0A024R2V1|A0A024R2V1_HUMAN	0.402	tr|A4UCS6|A4UCS6_HUMAN	0.609
tr|B2RA03|B2RA03_HUMAN	0.33	tr|B3VL28|B3VL28_HUMAN	0.403	tr|Q5T9B9|Q5T9B9_HUMAN	0.677
sp|P08727|K1C19_HUMAN	0.331	sp|P08727|K1C19_HUMAN	0.411	sp|P15848|ARSB_HUMAN	0.691
sp|A2RUH7|MBPHL_HUMAN	0.338	sp|P00918|CAH2_HUMAN	0.454	tr|V9GYG9|V9GYG9_HUMAN	0.704
tr|A0A024R2V1|A0A024R2V1_HUMAN	0.427	tr|B4DMR6|B4DMR6_HUMAN	0.458	tr|B4DX19|B4DX19_HUMAN	0.706
tr|B4DMR6|B4DMR6_HUMAN	0.43	tr|B2R6F7|B2R6F7_HUMAN	0.478	sp|Q9BW30|TPPP3_HUMAN	0.706
tr|B2R6F7|B2R6F7_HUMAN	0.444	sp|Q9Y623|MYH4_HUMAN	0.492	tr|A0A0A0MTR1|A0A0A0MTR1_HUMAN	0.708
tr|B3KUR3|B3KUR3_HUMAN	0.469	tr|B3KUR3|B3KUR3_HUMAN	0.545	tr|B4DGU4|B4DGU4_HUMAN	0.709
sp|P04040|CATA_HUMAN	0.538	sp|A2RUH7|MBPHL_HUMAN	0.552	tr|B4DL07|B4DL07_HUMAN	0.71
sp|P16157|ANK1_HUMAN	0.572	tr|A0A0A0MSG2|A0A0A0MSG2_HUMAN	0.581	sp|P09936|UCHL1_HUMAN	0.733
tr|B2R8P6|B2R8P6_HUMAN	0.576	sp|Q9UK22|FBX2_HUMAN	0.591	tr|C9JWC3|C9JWC3_HUMAN	0.736
tr|H3BN14|H3BN14_HUMAN	0.592	tr|B4DUL3|B4DUL3_HUMAN	0.608	tr|A0A075B6R9|A0A075B6R9_HUMAN	0.738
tr|A0A087WZE4|A0A087WZE4_HUMAN	0.615	tr|B7Z4A1|B7Z4A1_HUMAN	0.612	tr|A0A087X1J7|A0A087X1J7_HUMAN	0.738
tr|Q8N7G1|Q8N7G1_HUMAN	0.618	tr|K7ERI9|K7ERI9_HUMAN	0.626	sp|P19022|CADH2_HUMAN	0.745
sp|Q5T481|RBM20_HUMAN	0.622	sp|P16157|ANK1_HUMAN	0.643	sp|Q5BKX8|MURC_HUMAN	0.756

tr|A0A0A0MRE3|A0A0A0MRE3_HUMAN	0.622	tr|B4E1B2|B4E1B2_HUMAN	0.651	tr|K7ER74|K7ER74_HUMAN	0.757
tr|B7Z4V9|B7Z4V9_HUMAN	0.623	sp|Q9Y584|TIM22_HUMAN	0.66	sp|O75147|OBSL1_HUMAN	0.759
tr|Q0EFC6|Q0EFC6_HUMAN	0.629	sp|P09471|GNAO_HUMAN	0.661	sp|P01023|A2MG_HUMAN	0.761
sp|Q4G0N4|NAKD2_HUMAN	0.634	tr|B4DX19|B4DX19_HUMAN	0.662	tr|G3V1Y8|G3V1Y8_HUMAN	0.762
tr|A0A087WT59|A0A087WT59_HUMAN	0.636	tr|A0A024R2V4|A0A024R2V4_HUMAN	0.663	tr|S6AWE6|S6AWE6_HUMAN	0.768
sp|Q2TAY7|SMU1_HUMAN	0.638	tr|H6VRF8|H6VRF8_HUMAN	0.664	sp|P02792|FRIL_HUMAN	0.772
sp|P30043|BLVRB_HUMAN	0.649	sp|P04040|CATA_HUMAN	0.668	sp|P54652|HSP72_HUMAN	0.774
tr|A0A087WUF6|A0A087WUF6_HUMAN	0.65	sp|Q86TP1|PRUNE_HUMAN	0.669	tr|H0UI60|H0UI60_HUMAN	0.781
tr|I3L2B0|I3L2B0_HUMAN	0.663	tr|G3V1Y8|G3V1Y8_HUMAN	0.678	sp|A4UGR9|XIRP2_HUMAN	0.783
tr|A0A024R825|A0A024R825_HUMAN	0.664	tr|A0A024R4D5|A0A024R4D5_HUMAN	0.683	sp|Q9UJS0|CMC2_HUMAN	0.783
tr|B2RB32|B2RB32_HUMAN	0.664	tr|B2R8P6|B2R8P6_HUMAN	0.684	sp|P02144|MYG_HUMAN	0.784
sp|O00483|NDUA4_HUMAN	0.665	sp|P68133|ACTS_HUMAN	0.687	sp|Q9UJY1|HSPB8_HUMAN	0.787
sp|P12429|ANXA3_HUMAN	0.679	tr|A0A087WTK8|A0A087WTK8_HUMAN	0.689	tr|A8K141|A8K141_HUMAN	0.791
tr|H0YAC1|H0YAC1_HUMAN	0.681	tr|B4DL07|B4DL07_HUMAN	0.696	tr|A0A024RC87|A0A024RC87_HUMAN	0.791
sp|Q13228|SBP1_HUMAN	0.683	tr|A0A087X1J7|A0A087X1J7_HUMAN	0.696	tr|Q53FN1|Q53FN1_HUMAN	0.791
sp|Q5VTU8|AT5EL_HUMAN	0.685	tr|Q8N7G1|Q8N7G1_HUMAN	0.697	sp|Q99959|PKP2_HUMAN	0.793
sp|P62760|VISL1_HUMAN	0.686	sp|P30043|BLVRB_HUMAN	0.697	tr|A0A087WTE4|A0A087WTE4_HUMAN	0.796
sp|P26678|PPLA_HUMAN	0.696	tr|B4DDB3|B4DDB3_HUMAN	0.697	tr|A0A0A0MS87|A0A0A0MS87_HUMAN	0.798
tr|K7ERI9|K7ERI9_HUMAN	0.704	tr|A0A024R9B9|A0A024R9B9_HUMAN	0.699	tr|Q5UGI6|Q5UGI6_HUMAN	0.801
tr|H0YL90|H0YL90_HUMAN	0.708	sp|Q07507|DERM_HUMAN	0.701	tr|Q53GU8|Q53GU8_HUMAN	0.803
sp|P15090|FABP4_HUMAN	0.709	tr|B2RB32|B2RB32_HUMAN	0.704	tr|Q9HCC1|Q9HCC1_HUMAN	0.805
sp|P09455|RET1_HUMAN	0.71	sp|Q6UWY5|OLFL1_HUMAN	0.709	tr|A0A024R884|A0A024R884_HUMAN	0.805
sp|Q9NY47|CA2D2_HUMAN	0.714	sp|Q9NVI7|ATD3A_HUMAN	0.711	sp|P01619|KV301_HUMAN	0.807
tr|B4DVE1|B4DVE1_HUMAN	0.714	sp|Q9UBG0|MRC2_HUMAN	0.712	tr|E9PGC8|E9PGC8_HUMAN	0.809
tr|G3V325|G3V325_HUMAN	0.715	sp|Q5BKX8|MURC_HUMAN	0.712	sp|Q9Y676|RT18B_HUMAN	0.809

tr|B3KSZ1|B3KSZ1_HUMAN	0.716	tr|H3BM67|H3BM67_HUMAN	0.714	tr|H0Y8G4|H0Y8G4_HUMAN	0.811
sp|P09669|COX6C_HUMAN	0.717	tr|A0A087WZE4|A0A087WZE4_HUMAN	0.72	sp|Q6ZP80|TM182_HUMAN	0.812
tr|E7EQV9|E7EQV9_HUMAN	0.718	tr|A2RRE0|A2RRE0_HUMAN	0.722	tr|A0N5G5|A0N5G5_HUMAN	0.813
tr|H0YBX2|H0YBX2_HUMAN	0.718	tr|B3KM97|B3KM97_HUMAN	0.723	sp|P35221|CTNA1_HUMAN	0.817
tr|B2R6C0|B2R6C0_HUMAN	0.719	sp|O95210|STBD1_HUMAN	0.728	tr|Q6P528|Q6P528_HUMAN	0.818
tr|A0A0A0MR33|A0A0A0MR33_HUMAN	0.719	sp|Q2TAY7|SMU1_HUMAN	0.729	sp|Q06828|FMOD_HUMAN	0.821
tr|H7BXZ6|H7BXZ6_HUMAN	0.719	sp|Q05707|COEA1_HUMAN	0.733	tr|A5YM48|A5YM48_HUMAN	0.822
sp|P23141|EST1_HUMAN	0.72	sp|P23141|EST1_HUMAN	0.733	sp|Q86TP1|PRUNE_HUMAN	0.822
tr|H0YI09|H0YI09_HUMAN	0.721	sp|Q9H7C9|AAMDC_HUMAN	0.733	tr|K7EMC7|K7EMC7_HUMAN	0.822
tr|B4DMD3|B4DMD3_HUMAN	0.722	sp|P05154|IPSP_HUMAN	0.737	sp|Q8NDY3|ARHL1_HUMAN	0.823
tr|B7Z601|B7Z601_HUMAN	0.722	tr|B4DI75|B4DI75_HUMAN	0.738	tr|A0A0A0MQY0|A0A0A0MQY0_HUMAN	0.824
tr|D3DVH1|D3DVH1_HUMAN	0.726	tr|H0YL90|H0YL90_HUMAN	0.74	tr|A5PLL0|A5PLL0_HUMAN	0.824
sp|P61626|LYSC_HUMAN	0.728	tr|K7ER74|K7ER74_HUMAN	0.74	sp|Q07507|DERM_HUMAN	0.825
sp|Q14008|CKAP5_HUMAN	0.731	tr|B3KSZ1|B3KSZ1_HUMAN	0.741	sp|O60237|MYPT2_HUMAN	0.826
sp|P55001|MFAP2_HUMAN	0.731	tr|V9GYG9|V9GYG9_HUMAN	0.741	tr|H0YIV4|H0YIV4_HUMAN	0.826
tr|H3BUX2|H3BUX2_HUMAN	0.734	sp|Q9Y676|RT18B_HUMAN	0.741	sp|P02768|ALBU_HUMAN	0.827
sp|P05154|IPSP_HUMAN	0.737	tr|A0A024R172|A0A024R172_HUMAN	0.743	sp|Q9UBY9|HSPB7_HUMAN	0.828
tr|Q7Z3Z1|Q7Z3Z1_HUMAN	0.737	tr|B2R773|B2R773_HUMAN	0.743	sp|Q9NQC3|RTN4_HUMAN	0.83
tr|A0A024R248|A0A024R248_HUMAN	0.737	tr|U3KPS2|U3KPS2_HUMAN	0.745	sp|Q96DG6|CMBL_HUMAN	0.83
tr|Q6ZN40|Q6ZN40_HUMAN	0.738	tr|Q5VY30|Q5VY30_HUMAN	0.747	sp|O94811|TPPP_HUMAN	0.831
sp|P23142|FBLN1_HUMAN	0.739	tr|V9HW62|V9HW62_HUMAN	0.753	sp|Q9Y6I3|EPN1_HUMAN	0.831
tr|B1AP13|B1AP13_HUMAN	0.74	tr|J3KQY1|J3KQY1_HUMAN	0.755	tr|B4DFP1|B4DFP1_HUMAN	0.832
sp|Q9H7C9|AAMDC_HUMAN	0.74	sp|Q9UJS0|CMC2_HUMAN	0.755	tr|H0Y9H2|H0Y9H2_HUMAN	0.833
sp|P68133|ACTS_HUMAN	0.74	sp|P23142|FBLN1_HUMAN	0.756	tr|Q53GL5|Q53GL5_HUMAN	1.202
sp|Q9UBG0|MRC2_HUMAN	0.742	tr|A0A087X0E2|A0A087X0E2_HUMAN	0.756	tr|B4DWA5|B4DWA5_HUMAN	1.204
tr|H3BNX8|H3BNX8_HUMAN	0.746	tr|H3BN14|H3BN14_HUMAN	0.757	sp|Q96CN7|ISOC1_HUMAN	1.204
tr|E9PN17|E9PN17_HUMAN	0.747	tr|A0A087WXX9|A0A087WXX9_HUMAN	0.758	tr|B4DZG7|B4DZG7_HUMAN	1.204
tr|I3L0E3|I3L0E3_HUMAN	0.751	sp|P21397|AOFA_HUMAN	0.759	sp|Q9GZM7|TINAL_HUMAN	1.204

sp|O60240|PLIN1_HUMAN	0.753	tr|B4DNG0|B4DNG0_HUMAN	0.761	tr|J3KS22|J3KS22_HUMAN	1.205
tr|Q5VY30|Q5VY30_HUMAN	0.756	sp|Q7L311|ARMX2_HUMAN	0.761	sp|P23396|RS3_HUMAN	1.207
tr|A0A059U7G5|A0A059U7G5_HUMAN	0.757	sp|Q9HCB6|SPON1_HUMAN	0.763	tr|B0QYK0|B0QYK0_HUMAN	1.207
sp|Q4G176|ACSF3_HUMAN	0.758	tr|A0A024R825|A0A024R825_HUMAN	0.767	sp|P09669|COX6C_HUMAN	1.209
sp|P48047|ATPO_HUMAN	0.759	tr|B4DMD3|B4DMD3_HUMAN	0.772	tr|E7EQV9|E7EQV9_HUMAN	1.211
sp|P21397|AOFA_HUMAN	0.759	tr|A5PLL0|A5PLL0_HUMAN	0.773	sp|Q9NZM1|MYOF_HUMAN	1.212
tr|B4DX19|B4DX19_HUMAN	0.763	sp|P54652|HSP72_HUMAN	0.773	tr|B2R5W3|B2R5W3_HUMAN	1.212
tr|Q2TB59|Q2TB59_HUMAN	0.763	tr|B4DXZ6|B4DXZ6_HUMAN	0.774	tr|B0QYN7|B0QYN7_HUMAN	1.214
tr|A0A0A0MTQ5|A0A0A0MTQ5_HUMAN	0.764	sp|O00264|PGRC1_HUMAN	0.777	sp|P13010|XRCC5_HUMAN	1.215
tr|D3DWJ7|D3DWJ7_HUMAN	0.764	tr|A2RTY6|A2RTY6_HUMAN	0.777	sp|Q96IX5|USMG5_HUMAN	1.217
sp|P41222|PTGDS_HUMAN	0.766	tr|L8E853|L8E853_HUMAN	0.785	sp|Q92506|DHB8_HUMAN	1.218
sp|Q96IX5|USMG5_HUMAN	0.766	tr|H3BUX2|H3BUX2_HUMAN	0.788	tr|B7Z9B8|B7Z9B8_HUMAN	1.219
tr|A0A087X130|A0A087X130_HUMAN	0.768	tr|D3DXF2|D3DXF2_HUMAN	0.791	sp|Q8NBX0|SCPDL_HUMAN	1.22
sp|P21912|SDHB_HUMAN	0.772	sp|P43652|AFAM_HUMAN	0.795	tr|D3DVH1|D3DVH1_HUMAN	1.22
tr|A0A024R4D5|A0A024R4D5_HUMAN	0.772	sp|Q6ZP80|TM182_HUMAN	0.795	tr|A0A087WU72|A0A087WU72_HUMAN	1.221
sp|Q6UWY5|OLFL1_HUMAN	0.772	tr|A0A087X130|A0A087X130_HUMAN	0.796	tr|E2RVJ0|E2RVJ0_HUMAN	1.224
tr|Q9NSD0|Q9NSD0_HUMAN	0.775	tr|A0A024R2W4|A0A024R2W4_HUMAN	0.797	tr|H3BN14|H3BN14_HUMAN	1.227
sp|Q9NZ08|ERAP1_HUMAN	0.776	tr|B4DVE1|B4DVE1_HUMAN	0.8	tr|H0YMF4|H0YMF4_HUMAN	1.228
sp|P23368|MAOM_HUMAN	0.776	tr|A0A024R1X8|A0A024R1X8_HUMAN	0.801	tr|A6XNE2|A6XNE2_HUMAN	1.228
sp|Q13057|COASY_HUMAN	0.776	sp|Q9BX97|PLVAP_HUMAN	0.801	tr|A9LSU1|A9LSU1_HUMAN	1.23
sp|O00264|PGRC1_HUMAN	0.776	sp|P02768|ALBU_HUMAN	0.802	tr|A0A0A0MSZ4|A0A0A0MSZ4_HUMAN	1.234
tr|A8JZZ8|A8JZZ8_HUMAN	0.777	tr|B7Z5J1|B7Z5J1_HUMAN	0.803	sp|O76076|WISP2_HUMAN	1.238
sp|Q9BS92|NPS3B_HUMAN	0.777	tr|A5YM48|A5YM48_HUMAN	0.805	tr|A0A024R825|A0A024R825_HUMAN	1.238
tr|A0A024R9B9|A0A024R9B9_HUMAN	0.779	sp|P26678|PPLA_HUMAN	0.806	tr|E5RHP7|E5RHP7_HUMAN	1.242
tr|A0A087WT47|A0A087WT47_	0.781	tr|B4DGU4|B4DGU4_HUMAN	0.806	sp|Q03135|CAV1_HUMAN	1.249

HUMAN					
tr|A0A024R172|A0A024R172_HUMAN	0.786	tr|Q5T190|Q5T190_HUMAN	0.811	tr|A0A0A0MSQ0|A0A0A0MSQ0_HUMAN	1.251
tr|B4DZL5|B4DZL5_HUMAN	0.786	tr|S6BAR0|S6BAR0_HUMAN	0.812	tr|Q6IB58|Q6IB58_HUMAN	1.251
tr|B2RDE0|B2RDE0_HUMAN	0.788	sp|Q9BU61|NDUF3_HUMAN	0.82	sp|P12429|ANXA3_HUMAN	1.253
tr|B4E2J1|B4E2J1_HUMAN	0.789	sp|P05546|HEP2_HUMAN	0.821	sp|Q16853|AOC3_HUMAN	1.255
tr|Q53FC3|Q53FC3_HUMAN	0.792	sp|P52179|MYOM1_HUMAN	0.821	tr|A8K1D2|A8K1D2_HUMAN	1.256
sp|P82673|RT35_HUMAN	0.795	tr|A0A087WUF6|A0A087WUF6_HUMAN	0.821	tr|H0YG03|H0YG03_HUMAN	1.256
tr|B4DVV3|B4DVV3_HUMAN	0.796	tr|C9J712|C9J712_HUMAN	0.822	tr|E9PLM6|E9PLM6_HUMAN	1.258
sp|P14555|PA2GA_HUMAN	0.796	tr|B4DFP1|B4DFP1_HUMAN	0.824	sp|P10915|HPLN1_HUMAN	1.261
sp|O95210|STBD1_HUMAN	0.797	tr|B0AZQ4|B0AZQ4_HUMAN	0.825	tr|H9E7F4|H9E7F4_HUMAN	1.263
tr|A0A0A0MTR1|A0A0A0MTR1_HUMAN	0.797	sp|Q9H2M9|RBGPR_HUMAN	0.825	sp|Q8WWP7|GIMA1_HUMAN	1.263
tr|M0R0P7|M0R0P7_HUMAN	0.798	tr|B7Z8A2|B7Z8A2_HUMAN	0.829	tr|A0A024QZE7|A0A024QZE7_HUMAN	1.264
tr|A0A024R4Y7|A0A024R4Y7_HUMAN	0.798	sp|Q9HBL7|PLRKT_HUMAN	0.829	sp|Q9NZN4|EHD2_HUMAN	1.265
sp|Q8N5K1|CISD2_HUMAN	0.8	sp|Q6UXB8|PI16_HUMAN	0.83	sp|Q969Z3|MARC2_HUMAN	1.267
tr|B4DVZ0|B4DVZ0_HUMAN	0.801	tr|B4E2J1|B4E2J1_HUMAN	0.831	sp|P06899|H2B1J_HUMAN	1.268
tr|H0YHA7|H0YHA7_HUMAN	0.807	tr|A0A024R1E2|A0A024R1E2_HUMAN	0.831	sp|O95631|NET1_HUMAN	1.268
sp|Q07507|DERM_HUMAN	0.811	tr|A0A024R9U8|A0A024R9U8_HUMAN	0.832	tr|B2RA03|B2RA03_HUMAN	1.27
sp|P14854|CX6B1_HUMAN	0.813	sp|Q99959|PKP2_HUMAN	0.832	tr|Q6LAP8|Q6LAP8_HUMAN	1.273
tr|B3KUE5|B3KUE5_HUMAN	0.814	sp|O00483|NDUA4_HUMAN	0.832	tr|B4DTY8|B4DTY8_HUMAN	1.276
sp|A2RTX5|SYTC2_HUMAN	0.816	tr|B4DZL5|B4DZL5_HUMAN	0.832	tr|J3QRS3|J3QRS3_HUMAN	1.276
tr|A8K9B2|A8K9B2_HUMAN	0.816	tr|Q59HA3|Q59HA3_HUMAN	1.202	sp|P20700|LMNB1_HUMAN	1.279
sp|O75947|ATP5H_HUMAN	0.817	sp|Q06323|PSME1_HUMAN	1.203	tr|F2Z3A7|F2Z3A7_HUMAN	1.282
sp|Q9Y230|RUVB2_HUMAN	0.818	tr|M0QXK2|M0QXK2_HUMAN	1.203	tr|Q0D2M2|Q0D2M2_HUMAN	1.286
sp|P27338|AOFB_HUMAN	0.82	sp|P23381|SYWC_HUMAN	1.204	tr|B2RA94|B2RA94_HUMAN	1.292
sp|Q8WVV4|POF1B_HUMAN	0.82	tr|E7EVA0|E7EVA0_HUMAN	1.204	sp|Q8N5M1|ATPF2_HUMAN	1.293
tr|F8W1A4|F8W1A4_HUMAN	0.82	sp|P29218|IMPA1_HUMAN	1.206	tr|G3V1V0|G3V1V0_HUMAN	1.294
sp|Q9NPJ3|ACO13_HUMAN	0.821	sp|Q9NWV4|CA123_HUMAN	1.207	sp|O60240|PLIN1_HUMAN	1.296
tr|A0A024RBE8|A0A024RBE8_	0.821	tr|D6RGG3|D6RGG3_HUMAN	1.207	tr|B4E380|B4E380_HUMAN	1.296

HUMAN

tr|B4DNG0|B4DNG0_HUMAN	0.824	tr|A0A087WSV8|A0A087WSV8_HUMAN	1.207	sp|P02649|APOE_HUMAN	1.299
tr|B7Z5J1|B7Z5J1_HUMAN	0.824	sp|Q9UII2|ATIF1_HUMAN	1.207	sp|P08572|CO4A2_HUMAN	1.301
tr|Q5T8U5|Q5T8U5_HUMAN	0.824	tr|Q5CZH6|Q5CZH6_HUMAN	1.208	sp|P08729|K2C7_HUMAN	1.306
sp|Q8WW59|SPRY4_HUMAN	0.826	sp|P61604|CH10_HUMAN	1.209	sp|P00918|CAH2_HUMAN	1.308
sp|Q96FQ6|S10AG_HUMAN	0.827	sp|P61970|NTF2_HUMAN	1.209	sp|Q15417|CNN3_HUMAN	1.312
sp|P25325|THTM_HUMAN	0.828	sp|P07360|CO8G_HUMAN	1.212	sp|Q969G5|PRDBP_HUMAN	1.318
tr|A0A024RDX4|A0A024RDX4_HUMAN	0.829	tr|B3KSI7|B3KSI7_HUMAN	1.213	tr|Q6ZN40|Q6ZN40_HUMAN	1.322
sp|Q13425|SNTB2_HUMAN	0.829	tr|A0A024R258|A0A024R258_HUMAN	1.214	tr|Q5IWS5|Q5IWS5_HUMAN	1.322
sp|Q9BX97|PLVAP_HUMAN	0.83	sp|P05387|RLA2_HUMAN	1.214	tr|H0YGX7|H0YGX7_HUMAN	1.323
sp|P14927|QCR7_HUMAN	0.831	sp|A2RTX5|SYTC2_HUMAN	1.216	tr|B4E200|B4E200_HUMAN	1.337
sp|P43652|AFAM_HUMAN	0.832	tr|B2RDQ3|B2RDQ3_HUMAN	1.217	sp|Q15746|MYLK_HUMAN	1.346
tr|M0QYS1|M0QYS1_HUMAN	0.833	sp|Q5R372|RBG1L_HUMAN	1.217	tr|Q59FG9|Q59FG9_HUMAN	1.362
tr|K7ESP4|K7ESP4_HUMAN	0.833	tr|B3KM80|B3KM80_HUMAN	1.219	sp|P05109|S10A8_HUMAN	1.38
sp|P19652|A1AG2_HUMAN	1.201	sp|P62857|RS28_HUMAN	1.219	tr|B2ZGL7|B2ZGL7_HUMAN	1.381
tr|Q15374|Q15374_HUMAN	1.202	sp|P32418|NAC1_HUMAN	1.22	sp|P02042|HBD_HUMAN	1.385
tr|K7EKW4|K7EKW4_HUMAN	1.203	tr|B2RB23|B2RB23_HUMAN	1.22	tr|B7Z832|B7Z832_HUMAN	1.389
tr|Q5RKT7|Q5RKT7_HUMAN	1.203	tr|A0A090N8G0|A0A090N8G0_HUMAN	1.223	tr|A0A024QZJ6|A0A024QZJ6_HUMAN	1.393
tr|B4DS05|B4DS05_HUMAN	1.203	tr|B4E200|B4E200_HUMAN	1.224	tr|A0A024R035|A0A024R035_HUMAN	1.398
sp|P31946|1433B_HUMAN	1.203	sp|Q96QR8|PURB_HUMAN	1.224	sp|Q02338|BDH_HUMAN	1.417
sp|P01616|KV203_HUMAN	1.205	tr|H0YCP8|H0YCP8_HUMAN	1.225	sp|A2RTX5|SYTC2_HUMAN	1.425
tr|Q5U043|Q5U043_HUMAN	1.208	tr|J3KQ99|J3KQ99_HUMAN	1.225	sp|P49327|FAS_HUMAN	1.43
tr|B3KSI7|B3KSI7_HUMAN	1.208	tr|Q32Q10|Q32Q10_HUMAN	1.225	sp|Q01469|FABP5_HUMAN	1.436
tr|H0Y7A7|H0Y7A7_HUMAN	1.208	tr|B3KRM8|B3KRM8_HUMAN	1.226	tr|B4DEA7|B4DEA7_HUMAN	1.454
tr|Q53Y47|Q53Y47_HUMAN	1.211	sp|Q09028|RBBP4_HUMAN	1.227	tr|B2R941|B2R941_HUMAN	1.458
tr|G3V4P8|G3V4P8_HUMAN	1.211	tr|B4E0U6|B4E0U6_HUMAN	1.227	sp|O43707|ACTN4_HUMAN	1.473
sp|Q92890|UFD1_HUMAN	1.211	tr|H3BRU6|H3BRU6_HUMAN	1.23	tr|B3KUB8|B3KUB8_HUMAN	1.481
sp|P06727|APOA4_HUMAN	1.212	sp|Q8WUW1|BRK1_HUMAN	1.23	tr|B2R4M6|B2R4M6_HUMAN	1.487
tr|Q4G1C4|Q4G1C4_HUMAN	1.212	tr|Q3LIE9|Q3LIE9_HUMAN	1.231	tr|A0A024QZ34|A0A024QZ34_HUMAN	1.499

tr|A0A087WUQ6|A0A087WUQ6_HUMAN	1.213	sp|P26885|FKBP2_HUMAN	1.232	sp|P15121|ALDR_HUMAN	1.511
sp|Q15746|MYLK_HUMAN	1.213	sp|P26447|S10A4_HUMAN	1.232	tr|B4DVZ0|B4DVZ0_HUMAN	1.524
tr|E7EMG9|E7EMG9_HUMAN	1.214	sp|P0C0S5|H2AZ_HUMAN	1.232	sp|P05787|K2C8_HUMAN	1.531
tr|H3BPK3|H3BPK3_HUMAN	1.215	sp|O75368|SH3L1_HUMAN	1.233	tr|J3QRN6|J3QRN6_HUMAN	1.538
tr|V9HW38|V9HW38_HUMAN	1.215	sp|O00151|PDLI1_HUMAN	1.235	sp|P51911|CNN1_HUMAN	1.54
sp|Q9BX66|SRBS1_HUMAN	1.216	sp|Q15435|PP1R7_HUMAN	1.235	tr|E9PIM6|E9PIM6_HUMAN	1.545
sp|P37837|TALDO_HUMAN	1.216	tr|Q53Y47|Q53Y47_HUMAN	1.236	tr|G3XAP6|G3XAP6_HUMAN	1.549
sp|Q9H479|FN3K_HUMAN	1.218	tr|Q15374|Q15374_HUMAN	1.236	sp|P59665|DEF1_HUMAN	1.551
sp|Q04917|1433F_HUMAN	1.22	tr|A9LSU1|A9LSU1_HUMAN	1.237	sp|A2RUH7|MBPHL_HUMAN	1.574
sp|Q15843|NEDD8_HUMAN	1.22	tr|B2R6C0|B2R6C0_HUMAN	1.239	sp|P31146|COR1A_HUMAN	1.61
tr|A0A0A0MSG2|A0A0A0MSG2_HUMAN	1.221	sp|Q9Y5S9|RBM8A_HUMAN	1.241	sp|P08493|MGP_HUMAN	1.676
tr|Q59EG0|Q59EG0_HUMAN	1.223	sp|O95433|AHSA1_HUMAN	1.243	tr|B3KVC9|B3KVC9_HUMAN	1.68
sp|Q96JB5|CK5P3_HUMAN	1.225	sp|P13929|ENOB_HUMAN	1.244	tr|B3KTQ2|B3KTQ2_HUMAN	1.685
tr|B2R4M6|B2R4M6_HUMAN	1.226	tr|B3KX72|B3KX72_HUMAN	1.244	sp|Q9NR12|PDLI7_HUMAN	1.695
sp|P13929|ENOB_HUMAN	1.231	sp|P43490|NAMPT_HUMAN	1.245	tr|I1VZV6|I1VZV6_HUMAN	1.76
sp|P61158|ARP3_HUMAN	1.232	sp|P68036|UB2L3_HUMAN	1.245	tr|B2R6C0|B2R6C0_HUMAN	1.768
sp|O15144|ARPC2_HUMAN	1.233	tr|A0A087WXM6|A0A087WXM6_HUMAN	1.247	sp|P02747|C1QC_HUMAN	1.782
sp|P35579|MYH9_HUMAN	1.234	tr|B3KM95|B3KM95_HUMAN	1.247	sp|O75339|CILP1_HUMAN	1.816
sp|Q9UJY1|HSPB8_HUMAN	1.234	sp|O75369|FLNB_HUMAN	1.247	tr|B3KRN4|B3KRN4_HUMAN	1.931
tr|A0A090N8G0|A0A090N8G0_HUMAN	1.236	tr|Q6FIE5|Q6FIE5_HUMAN	1.248	sp|P61626|LYSC_HUMAN	1.949
tr|B5BTY4|B5BTY4_HUMAN	1.239	tr|A8K4G7|A8K4G7_HUMAN	1.249	sp|P02743|SAMP_HUMAN	2.128
sp|Q16082|HSPB2_HUMAN	1.239	sp|Q13103|SPP24_HUMAN	1.249	tr|X6RLJ0|X6RLJ0_HUMAN	2.168
sp|Q13442|HAP28_HUMAN	1.239	tr|B3KTA3|B3KTA3_HUMAN	1.251	sp|Q8N474|SFRP1_HUMAN	2.428
sp|P19623|SPEE_HUMAN	1.24	sp|Q9NR28|DBLOH_HUMAN	1.251	sp|P48061|SDF1_HUMAN	2.448
sp|Q9UMX5|NENF_HUMAN	1.241	sp|Q15293|RCN1_HUMAN	1.251	tr|A1KY36|A1KY36_HUMAN	2.539
sp|P63104|1433Z_HUMAN	1.243	sp|Q96AG4|LRC59_HUMAN	1.255	sp|P15090|FABP4_HUMAN	2.616
sp|Q4KWH8|PLCH1_HUMAN	1.246	tr|A0A024R5M3|A0A024R5M3_HUMAN	1.256		
sp|P62820|RAB1A_HUMAN	1.249	tr|M0QYT0|M0QYT0_HUMAN	1.257		
sp|Q9BW30|TPPP3_HUMAN	1.249	tr|B3KN06|B3KN06_HUMAN	1.257		
sp|Q13103|SPP24_HUMAN	1.25	sp|Q9NZA1|CLIC5_HUMAN	1.258		

sp|O15061|SYNEM_HUMAN	1.25	tr|H0Y614|H0Y614_HUMAN	1.258
tr|A0A024QZT9|A0A024QZT9_HUMAN	1.251	tr|A0A087WYR3|A0A087WYR3_HUMAN	1.26
sp|Q9NP98|MYOZ1_HUMAN	1.254	tr|J3KR44|J3KR44_HUMAN	1.26
sp|Q02252|MMSA_HUMAN	1.255	tr|X6RFL8|X6RFL8_HUMAN	1.26
sp|O95393|BMP10_HUMAN	1.255	sp|Q9UMX5|NENF_HUMAN	1.261
sp|Q15435|PP1R7_HUMAN	1.255	tr|B2RAY1|B2RAY1_HUMAN	1.261
sp|P31146|COR1A_HUMAN	1.257	sp|O43776|SYNC_HUMAN	1.261
tr|A0A087WTH0|A0A087WTH0_HUMAN	1.257	sp|Q96JB5|CK5P3_HUMAN	1.262
tr|M0QXF9|M0QXF9_HUMAN	1.258	sp|P31946|1433B_HUMAN	1.263
sp|Q9GZM7|TINAL_HUMAN	1.258	sp|Q03135|CAV1_HUMAN	1.264
sp|P09211|GSTP1_HUMAN	1.259	tr|B4DQ92|B4DQ92_HUMAN	1.265
tr|B4DH02|B4DH02_HUMAN	1.26	tr|B3KXD1|B3KXD1_HUMAN	1.268
tr|E9PB61|E9PB61_HUMAN	1.261	tr|Q9UL89|Q9UL89_HUMAN	1.269
sp|P07737|PROF1_HUMAN	1.261	tr|Q53FI7|Q53FI7_HUMAN	1.269
tr|Q6FIE5|Q6FIE5_HUMAN	1.262	sp|Q4KWH8|PLCH1_HUMAN	1.271
tr|D6RAW2|D6RAW2_HUMAN	1.262	tr|B4DVZ0|B4DVZ0_HUMAN	1.273
sp|P19022|CADH2_HUMAN	1.266	sp|P61586|RHOA_HUMAN	1.273
tr|A0A0A0MQY0|A0A0A0MQY0_HUMAN	1.271	tr|A0A0A0MTH3|A0A0A0MTH3_HUMAN	1.278
tr|A0A0A0MSQ0|A0A0A0MSQ0_HUMAN	1.271	tr|Q5U071|Q5U071_HUMAN	1.279
sp|Q14315|FLNC_HUMAN	1.271	tr|Q5U043|Q5U043_HUMAN	1.28
tr|A0A087WYR3|A0A087WYR3_HUMAN	1.273	tr|A0A087X1K9|A0A087X1K9_HUMAN	1.28
sp|P26038|MOES_HUMAN	1.275	sp|Q96D15|RCN3_HUMAN	1.28
tr|J3KQ99|J3KQ99_HUMAN	1.278	tr|B4E2A3|B4E2A3_HUMAN	1.284
tr|A1L172|A1L172_HUMAN	1.279	sp|Q02252|MMSA_HUMAN	1.284
sp|Q9UBY9|HSPB7_HUMAN	1.28	tr|A0A087WTH0|A0A087WTH0_HUMAN	1.284
sp|P35754|GLRX1_HUMAN	1.282	tr|B2R5W3|B2R5W3_HUMAN	1.286
tr|Q8TCF0|Q8TCF0_HUMAN	1.283	tr|B4DWC4|B4DWC4_HUMAN	1.286
sp|Q9H987|SYP2L_HUMAN	1.284	tr|Q5JNW7|Q5JNW7_HUMAN	1.287
sp|P07305|H10_HUMAN	1.284	tr|Q5RKT7|Q5RKT7_HUMAN	1.291

tr|A0A024R895|A0A024R895_HUMAN	1.286	sp|Q0ZGT2|NEXN_HUMAN	1.291
sp|Q8WW22|DNJA4_HUMAN	1.288	tr|Q6P528|Q6P528_HUMAN	1.291
sp|Q01995|TAGL_HUMAN	1.288	tr|A0A024R9E2|A0A024R9E2_HUMAN	1.292
tr|G3V1V0|G3V1V0_HUMAN	1.292	sp|P19021|AMD_HUMAN	1.296
sp|Q13885|TBB2A_HUMAN	1.292	tr|B4DWA5|B4DWA5_HUMAN	1.296
tr|B3KXC3|B3KXC3_HUMAN	1.293	sp|Q04917|1433F_HUMAN	1.299
sp|Q01469|FABP5_HUMAN	1.293	sp|Q8WW22|DNJA4_HUMAN	1.3
sp|Q9HCH3|CPNE5_HUMAN	1.293	tr|H0Y9H2|H0Y9H2_HUMAN	1.301
tr|B3KXD1|B3KXD1_HUMAN	1.294	tr|H0YL18|H0YL18_HUMAN	1.301
tr|C9JC84|C9JC84_HUMAN	1.299	sp|P52907|CAZA1_HUMAN	1.301
tr|B5BUI8|B5BUI8_HUMAN	1.3	sp|P62820|RAB1A_HUMAN	1.303
tr|A0A024R091|A0A024R091_HUMAN	1.303	sp|Q02338|BDH_HUMAN	1.305
tr|B2R6M6|B2R6M6_HUMAN	1.303	tr|B2R6S5|B2R6S5_HUMAN	1.305
sp|O60237|MYPT2_HUMAN	1.305	tr|A0A024R091|A0A024R091_HUMAN	1.306
tr|F8WAR2|F8WAR2_HUMAN	1.305	tr|A8K4V6|A8K4V6_HUMAN	1.306
tr|H7BZJ3|H7BZJ3_HUMAN	1.308	sp|Q9BX66|SRBS1_HUMAN	1.308
sp|P78539|SRPX_HUMAN	1.309	tr|B3KR50|B3KR50_HUMAN	1.308
tr|Q59GM9|Q59GM9_HUMAN	1.31	sp|P31949|S10AB_HUMAN	1.309
tr|H0Y614|H0Y614_HUMAN	1.311	tr|H0YIV4|H0YIV4_HUMAN	1.31
tr|B2RAY1|B2RAY1_HUMAN	1.312	sp|Q9BRA2|TXD17_HUMAN	1.31
tr|B4DV28|B4DV28_HUMAN	1.316	tr|B5BU25|B5BU25_HUMAN	1.313
sp|P02671|FIBA_HUMAN	1.322	sp|P07237|PDIA1_HUMAN	1.313
tr|B4DWC4|B4DWC4_HUMAN	1.327	tr|H3BPE7|H3BPE7_HUMAN	1.318
tr|A0A087WZF1|A0A087WZF1_HUMAN	1.327	tr|A0A087WXL8|A0A087WXL8_HUMAN	1.32
sp|P07437|TBB5_HUMAN	1.336	tr|B4DNP0|B4DNP0_HUMAN	1.321
sp|Q07960|RHG01_HUMAN	1.337	tr|C9J8S2|C9J8S2_HUMAN	1.323
tr|A0A068LKR7|A0A068LKR7_HUMAN	1.34	sp|P07305|H10_HUMAN	1.326
tr|B2R6D7|B2R6D7_HUMAN	1.347	sp|O15144|ARPC2_HUMAN	1.327
tr|Q5U000|Q5U000_HUMAN	1.348	tr|E7EQ72|E7EQ72_HUMAN	1.327

sp|P35221|CTNA1_HUMAN	1.348	sp|Q16543|CDC37_HUMAN	1.328
tr|B2RDY9|B2RDY9_HUMAN	1.349	sp|Q702N8|XIRP1_HUMAN	1.33
tr|E7EVA0|E7EVA0_HUMAN	1.349	tr|J3KS22|J3KS22_HUMAN	1.334
sp|P09936|UCHL1_HUMAN	1.356	sp|P31150|GDIA_HUMAN	1.338
tr|A0A024RDA6|A0A024RDA6_HUMAN	1.357	tr|B4DV28|B4DV28_HUMAN	1.339
sp|Q15847|ADIRF_HUMAN	1.361	sp|P20962|PTMS_HUMAN	1.339
tr|B3KQK4|B3KQK4_HUMAN	1.363	sp|Q9Y266|NUDC_HUMAN	1.34
sp|Q702N8|XIRP1_HUMAN	1.364	tr|Q7KZA3|Q7KZA3_HUMAN	1.343
sp|O94811|TPPP_HUMAN	1.366	sp|P26038|MOES_HUMAN	1.343
tr|B4DEA6|B4DEA6_HUMAN	1.369	sp|P61158|ARP3_HUMAN	1.344
sp|P02763|A1AG1_HUMAN	1.369	tr|Q6DEN2|Q6DEN2_HUMAN	1.345
sp|Q9BUF5|TBB6_HUMAN	1.373	sp|P23396|RS3_HUMAN	1.345
tr|A4UCS6|A4UCS6_HUMAN	1.374	tr|A0A024RDB4|A0A024RDB4_HUMAN	1.345
tr|E9PGC8|E9PGC8_HUMAN	1.374	sp|Q15181|IPYR_HUMAN	1.347
sp|Q9UDY4|DNJB4_HUMAN	1.376	sp|P01034|CYTC_HUMAN	1.35
sp|P37802|TAGL2_HUMAN	1.381	sp|P50897|PPT1_HUMAN	1.352
sp|Q9BXX0|EMIL2_HUMAN	1.383	sp|O15511|ARPC5_HUMAN	1.359
tr|E5KN59|E5KN59_HUMAN	1.384	sp|Q9NZU5|LMCD1_HUMAN	1.364
tr|A8K3B0|A8K3B0_HUMAN	1.386	tr|A0A024R895|A0A024R895_HUMAN	1.366
sp|P04080|CYTB_HUMAN	1.388	tr|F8WCF6|F8WCF6_HUMAN	1.368
sp|Q9UHL4|DPP2_HUMAN	1.39	sp|P02763|A1AG1_HUMAN	1.37
tr|A0A024R6X1|A0A024R6X1_HUMAN	1.392	sp|P07437|TBB5_HUMAN	1.373
sp|Q9NR12|PDLI7_HUMAN	1.393	tr|B4DKJ4|B4DKJ4_HUMAN	1.384
sp|P15848|ARSB_HUMAN	1.393	tr|J3KRG9|J3KRG9_HUMAN	1.386
tr|Q9UL89|Q9UL89_HUMAN	1.4	tr|B2R6M6|B2R6M6_HUMAN	1.387
sp|Q0ZGT2|NEXN_HUMAN	1.409	tr|A1L172|A1L172_HUMAN	1.396
sp|P01619|KV301_HUMAN	1.415	tr|B2RDY9|B2RDY9_HUMAN	1.397
tr|B2R548|B2R548_HUMAN	1.416	tr|B4DND4|B4DND4_HUMAN	1.4
sp|Q9NZA1|CLIC5_HUMAN	1.423	tr|A0A024RAM4|A0A024RAM4_HUMAN	1.402
sp|O95817|BAG3_HUMAN	1.426	tr|A0A024RDA6|A0A024RDA6	1.403

		_HUMAN	
tr|J3KTF8|J3KTF8_HUMAN	1.431	sp|P08294|SODE_HUMAN	1.406
sp|P20962|PTMS_HUMAN	1.433	tr|A0A087X1Z3|A0A087X1Z3_HUMAN	1.409
sp|P00488|F13A_HUMAN	1.437	sp|P62306|RUXF_HUMAN	1.412
tr|A0A024RAF2|A0A024RAF2_HUMAN	1.437	tr|M0QXF9|M0QXF9_HUMAN	1.417
sp|Q14894|CRYM_HUMAN	1.441	sp|P06727|APOA4_HUMAN	1.417
sp|Q9NZN3|EHD3_HUMAN	1.444	tr|A6XNE2|A6XNE2_HUMAN	1.427
tr|B4DKJ4|B4DKJ4_HUMAN	1.447	tr|J3KPM9|J3KPM9_HUMAN	1.429
tr|A0A024R493|A0A024R493_HUMAN	1.45	tr|K7EMY7|K7EMY7_HUMAN	1.434
sp|P27348|1433T_HUMAN	1.451	tr|V9HW38|V9HW38_HUMAN	1.435
sp|P01903|DRA_HUMAN	1.461	sp|P14550|AK1A1_HUMAN	1.438
sp|P31150|GDIA_HUMAN	1.464	tr|A8K3B0|A8K3B0_HUMAN	1.439
sp|Q15417|CNN3_HUMAN	1.473	tr|B5BUI8|B5BUI8_HUMAN	1.44
tr|G3V3X5|G3V3X5_HUMAN	1.478	tr|Q8TCF0|Q8TCF0_HUMAN	1.442
sp|Q15942|ZYX_HUMAN	1.484	tr|B0QYK0|B0QYK0_HUMAN	1.444
tr|B4DUJ6|B4DUJ6_HUMAN	1.484	tr|Q6N093|Q6N093_HUMAN	1.445
sp|P12109|CO6A1_HUMAN	1.486	sp|O95631|NET1_HUMAN	1.445
tr|A0A087WUI4|A0A087WUI4_HUMAN	1.494	sp|P00338|LDHA_HUMAN	1.447
sp|P12110|CO6A2_HUMAN	1.498	sp|P78539|SRPX_HUMAN	1.448
tr|H0YIV4|H0YIV4_HUMAN	1.508	tr|B4E3A8|B4E3A8_HUMAN	1.451
tr|B4E3A8|B4E3A8_HUMAN	1.51	tr|A0A024R493|A0A024R493_HUMAN	1.465
tr|B2R6S5|B2R6S5_HUMAN	1.51	sp|P37802|TAGL2_HUMAN	1.467
tr|B3KXB8|B3KXB8_HUMAN	1.516	sp|P61626|LYSC_HUMAN	1.471
tr|Q6P528|Q6P528_HUMAN	1.517	tr|E7EMG9|E7EMG9_HUMAN	1.472
tr|A2A2D0|A2A2D0_HUMAN	1.523	sp|Q9NZN3|EHD3_HUMAN	1.478
tr|B4DI69|B4DI69_HUMAN	1.527	tr|A0A024R0K2|A0A024R0K2_HUMAN	1.485
tr|J3KRG9|J3KRG9_HUMAN	1.536	tr|J3QRS3|J3QRS3_HUMAN	1.491
tr|Q5STX8|Q5STX8_HUMAN	1.55	tr|B3KM68|B3KM68_HUMAN	1.496
sp|Q6EEV6|SUMO4_HUMAN	1.553	tr|W6A4U0|W6A4U0_HUMAN	1.498

sp|P0C0L5|CO4B_HUMAN	1.566
sp|P08311|CATG_HUMAN	1.59
tr|Q53FB0|Q53FB0_HUMAN	1.601
tr|Q53FB6|Q53FB6_HUMAN	1.609
sp|P67936|TPM4_HUMAN	1.62
tr|B4DEX8|B4DEX8_HUMAN	1.622
sp|P50461|CSRP3_HUMAN	1.622
tr|Q3B874|Q3B874_HUMAN	1.65
sp|P51911|CNN1_HUMAN	1.651
tr|W6A4U0|W6A4U0_HUMAN	1.661
tr|Q6N093|Q6N093_HUMAN	1.664
tr|B4DL49|B4DL49_HUMAN	1.708
sp|P18065|IBP2_HUMAN	1.765
tr|H0YGX7|H0YGX7_HUMAN	1.793
tr|A0A024QZX5|A0A024QZX5_HUMAN	1.804
sp|Q8TAE6|PP14C_HUMAN	1.808
tr|H0Y5J4|H0Y5J4_HUMAN	1.857
tr|A0A075B6R9|A0A075B6R9_HUMAN	2.071
tr|H3BRN4|H3BRN4_HUMAN	2.089
tr|C9JWC3|C9JWC3_HUMAN	2.149
tr|B7Z6G4|B7Z6G4_HUMAN	2.151
tr|A0A5E4|A0A5E4_HUMAN	2.432
tr|A0A024R884|A0A024R884_HUMAN	2.603

tr|B4DI69|B4DI69_HUMAN	1.498
tr|B5BUB5|B5BUB5_HUMAN	1.499
sp|P27348|1433T_HUMAN	1.505
sp|Q07960|RHG01_HUMAN	1.508
sp|P04080|CYTB_HUMAN	1.51
tr|B4DEA6|B4DEA6_HUMAN	1.511
tr|A8K1D2|A8K1D2_HUMAN	1.512
sp|P07737|PROF1_HUMAN	1.514
sp|P08493|MGP_HUMAN	1.518
sp|P0C0L5|CO4B_HUMAN	1.522
tr|A0A087WZF1|A0A087WZF1_HUMAN	1.523
tr|B2R941|B2R941_HUMAN	1.535
tr|E5KN59|E5KN59_HUMAN	1.537
sp|Q6EEV6|SUMO4_HUMAN	1.543
tr|A0A087WUS0|A0A087WUS0_HUMAN	1.558
sp|P15121|ALDR_HUMAN	1.56
tr|B4DVR4|B4DVR4_HUMAN	1.56
tr|A0A024RAF2|A0A024RAF2_HUMAN	1.563
tr|A2A2D0|A2A2D0_HUMAN	1.568
sp|O75339|CILP1_HUMAN	1.568
tr|Q53FB0|Q53FB0_HUMAN	1.572
tr|B3KXB8|B3KXB8_HUMAN	1.579
tr|B4DUJ6|B4DUJ6_HUMAN	1.581
tr|A0A0A0MSQ0|A0A0A0MSQ0_HUMAN	1.588
tr|A0A024QZJ6|A0A024QZJ6_HUMAN	1.593
sp|P12111|CO6A3_HUMAN	1.594
tr|A0A075B6R9|A0A075B6R9_HUMAN	1.609
tr|C9JWC3|C9JWC3_HUMAN	1.61

sp|P01903|DRA_HUMAN	1.614
tr|J3KTF8|J3KTF8_HUMAN	1.618
tr|Q7Z5G3|Q7Z5G3_HUMAN	1.638
sp|P02747|C1QC_HUMAN	1.643
tr|Q53FB6|Q53FB6_HUMAN	1.662
sp|Q15942|ZYX_HUMAN	1.669
tr|B3KUB8|B3KUB8_HUMAN	1.713
tr|B4DL49|B4DL49_HUMAN	1.728
sp|P05109|S10A8_HUMAN	1.739
sp|P00488|F13A_HUMAN	1.751
sp|P67936|TPM4_HUMAN	1.755
sp|O43707|ACTN4_HUMAN	1.772
sp|P35579|MYH9_HUMAN	1.784
sp|Q969G5|PRDBP_HUMAN	1.79
tr|B7Z832|B7Z832_HUMAN	1.799
sp|Q15746|MYLK_HUMAN	1.803
tr|B2R4M6|B2R4M6_HUMAN	1.817
sp|P12110|CO6A2_HUMAN	1.828
tr|A0A024QZX5|A0A024QZX5_HUMAN	1.853
sp|Q01469|FABP5_HUMAN	1.854
sp|P04004|VTNC_HUMAN	1.864
tr|E9PIM6|E9PIM6_HUMAN	1.877
sp|P50461|CSRP3_HUMAN	1.883
tr|G3V1V0|G3V1V0_HUMAN	1.959
tr|A0A0A0MSV6|A0A0A0MSV6_HUMAN	1.97
tr|G3V3X5|G3V3X5_HUMAN	1.996
sp|Q15417|CNN3_HUMAN	2.025
tr|A0A087X094|A0A087X094_HUMAN	2.051
sp|P48061|SDF1_HUMAN	2.057
tr|B3KVC9|B3KVC9_HUMAN	2.071
tr|Q6IBG1|Q6IBG1_HUMAN	2.119
tr|A0A024R884|A0A024R884_H	2.16

UMAN	
sp|P31146|COR1A_HUMAN	2.232
tr|A0A5E4|A0A5E4_HUMAN	2.315
tr|B3KRN4|B3KRN4_HUMAN	2.343
tr|A0A087WUI4|A0A087WUI4_HUMAN	2.402
sp|P18065|IBP2_HUMAN	2.402
tr|H0YGX7|H0YGX7_HUMAN	2.451
tr|X6RLJ0|X6RLJ0_HUMAN	2.638
sp|Q8N474|SFRP1_HUMAN	2.758
tr|B7Z6G4|B7Z6G4_HUMAN	2.772
sp|P51911|CNN1_HUMAN	2.881
